# Investigating the Effectiveness of an Enhanced Coin-Shaped Lithium Battery With Titanium Coating in Reducing Esophageal Damage From Accidental Ingestion in Living Piglets

**DOI:** 10.7759/cureus.89332

**Published:** 2025-08-04

**Authors:** Shinsuke Ohashi, Daisuke Kanamori, Tadayoshi Takahashi, Tomohiro Yagishita, Goki Uchida, Kazuaki Miyaguni, Tetsuro Sugihara, Ai Iwauchi, Mari Satake, Nei Fukasawa, Masashi Kurobe, Takao Ohki

**Affiliations:** 1 Pediatric Surgery, The Jikei University School of Medicine, Tokyo, JPN; 2 Energy Device Business Division, Industrial Energy Business Unit, Cell Development Department, Panasonic Energy Co. Ltd., Osaka, JPN; 3 Pathology, The Jikei University School of Medicine, Tokyo, JPN; 4 Vascular Surgery, The Jikei University School of Medicine, Tokyo, JPN

**Keywords:** button battery ingestion, coin-shaped lithium battery, electrochemical reaction, esophageal tissue damage, saline soak test, titanium-clad

## Abstract

Background

Accidental ingestion of coin-shaped lithium batteries (CSLBs) poses a serious health risk, leading to severe esophageal injuries and fatal complications. Conventional CSLBs cause rapid tissue damage due to electrochemical reactions, necessitating the development of safer battery designs. This study aimed to develop and evaluate an improved CSLB with a titanium-clad design to reduce electrochemical reactions and delay esophageal tissue damage in cases of accidental ingestion.

Methods

This study aimed to develop an improved CSLB using a three-layer clad structure (nickel-titanium-stainless steel) to reduce electrochemical reactions and delay tissue damage. A saline soak test was conducted to simulate battery ingestion and measure electrochemical activity. Additionally, an in vivo piglet experiment was used to assess the extent of esophageal tissue damage over time, comparing the improved battery with conventional CSLBs.

Results

The improved battery exhibited a significantly reduced electrochemical reaction, with a one-third lower consumed capacity in the saline soak test compared with conventional CSLBs. In vivo experiments demonstrated that full-thickness esophageal damage was delayed by four to six times, extending the time window for medical intervention from 2 h to 12 h. No perforation of the positive pole casing was observed in the improved battery compared with conventional CSLBs, indicating a safer design.

Conclusion

The novel titanium-clad CSLB successfully mitigates esophageal damage, providing critical additional time for emergency intervention. Although the improved design does not completely prevent injury, it significantly reduces the risk of fatal complications. These findings support the adoption of safer battery designs and the use of standardized test methods for future battery safety evaluations.

## Introduction

Accidental ingestion of coin-shaped batteries has been associated with severe complications such as tracheoesophageal and aortoesophageal fistulas. Reports from the National Poison Data System and the National Battery Ingestion Hotline in the United States reveal that between 1985 and 2017, the annual incidence of accidental ingestion of coin-shaped and button-shaped batteries ranged from 6 to 15 cases per million people. Although the incidence of fatalities and severe complications was below 1% in 1985, this figure gradually increased, surging in 2006 and exceeding 4% by 2017 [1]. A notable feature of coin-shaped lithium batteries (CSLBs) is their larger diameter compared with button-shaped alkaline batteries. In our study of battery ingestion incidents in Japan, we found that severe complications occurred in only 0.1% of cases involving button-shaped alkaline batteries. In contrast, the rate was significantly higher, at 10.5%, for CSLBs [2]. The International Electrotechnical Commission (IEC) has implemented safety measures for coin-shaped batteries with diameters of ≥16 mm, including child-resistant packaging and the engraving of pictograms on the outer case of the battery’s positive pole.

In a previous study using a live piglet model, we investigated the effects of ingesting CSLBs and reported that esophageal tissue damage progresses over time. Specifically, we observed perforations appearing in the outer case of the battery’s positive pole after 6 h, leading to a rapid exacerbation of tissue damage [3]. This damage is caused by the leakage of electrolytes and the reaction between water that infiltrates the battery and its positive and negative pole materials (manganese dioxide and lithium), generating additional alkaline substances. In collaboration with a battery manufacturer (Panasonic Energy Co., Ltd.), we aimed to develop a new battery that would reduce esophageal damage in case of accidental ingestion. Our objectives include creating a battery with a slower reaction rate to provide more time for battery removal and tissue damage minimization.

To reduce the occurrence of accidental ingestion of coin-shaped batteries, efforts are being made to develop technologies that mitigate their harmful effects on the human body. However, no standardized method currently exists for quantitatively assessing the extent of biological damage, which has hindered progress in technological development. The IEC is working on establishing regulations to reduce battery ingestion incidents; however, a standardized evaluation test for simulating battery ingestion is lacking.

An experiment was conducted using the improved battery, following the same methodology as described in the previous study [3]. The evaluation focused on whether the improved battery developed small perforations, whether electrolyte leakage occurred, and to what extent the histological response in living tissue could be delayed. Additionally, the relationship between reaction capacity in the simulated test and the in vivo experiment was examined.

## Materials and methods

Improved battery

**Figure 1:**
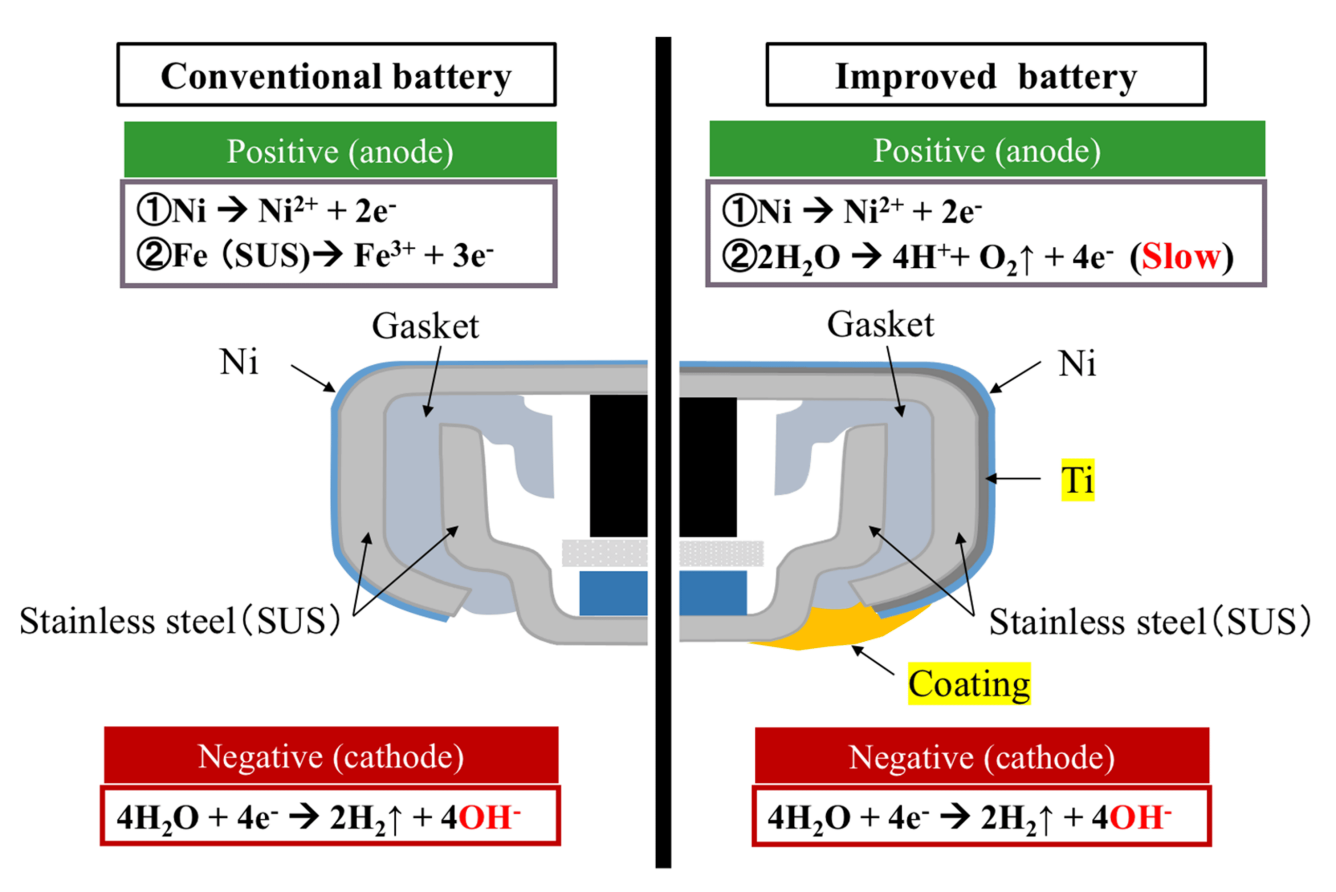
Structure of conventional and improved battery The titanium layer protects the dissolving of the stainless steel. The coating between the positive and negative poles protects the outer case edge of the positive pole and prevents the stainless steel from dissolution. Image Credits: Tadayoshi Takahashi

In conventional lithium batteries, the outer stainless-steel layer is coated with nickel plating. However, since titanium is a difficult material to plate, the improved battery adopted a three-layer clad structure, with titanium inserted between the stainless steel and nickel layers (Figure 1).

The thickness of the metals in the outer case on the positive pole and the final external dimensions of the battery remained the same as those of conventional products. Although titanium is non-magnetic, the inclusion of a stainless-steel layer ensures compatibility with magnet catheters, facilitating battery removal in cases of accidental ingestion. Furthermore, a resin coating was applied between the positive and negative outer cases to prevent exposure of the stainless steel layer at the edge of the positive outer case [4].

To ensure that the battery's performance was not compromised, its capacity was measured by connecting a 15 kΩ resistor, as recommended by the IEC [5].

Saline soak test

The saline soak test was devised to simulate CSLB ingestion. The test was carried out at an ambient temperature of 20 ± 5 °C, following a systematic approach outlined below:

A new piece of filter paper (diameter of 55 mm, thickness of 0.20 mm ± 0.04 mm, and surface density of 100 g/m^2^ ± 20 g/m^2^) was placed in a clean and dry Petri dish (inner diameter of 69 mm, lid with outer diameter of 80 mm; laboratory glassware).

**Figure 2:**
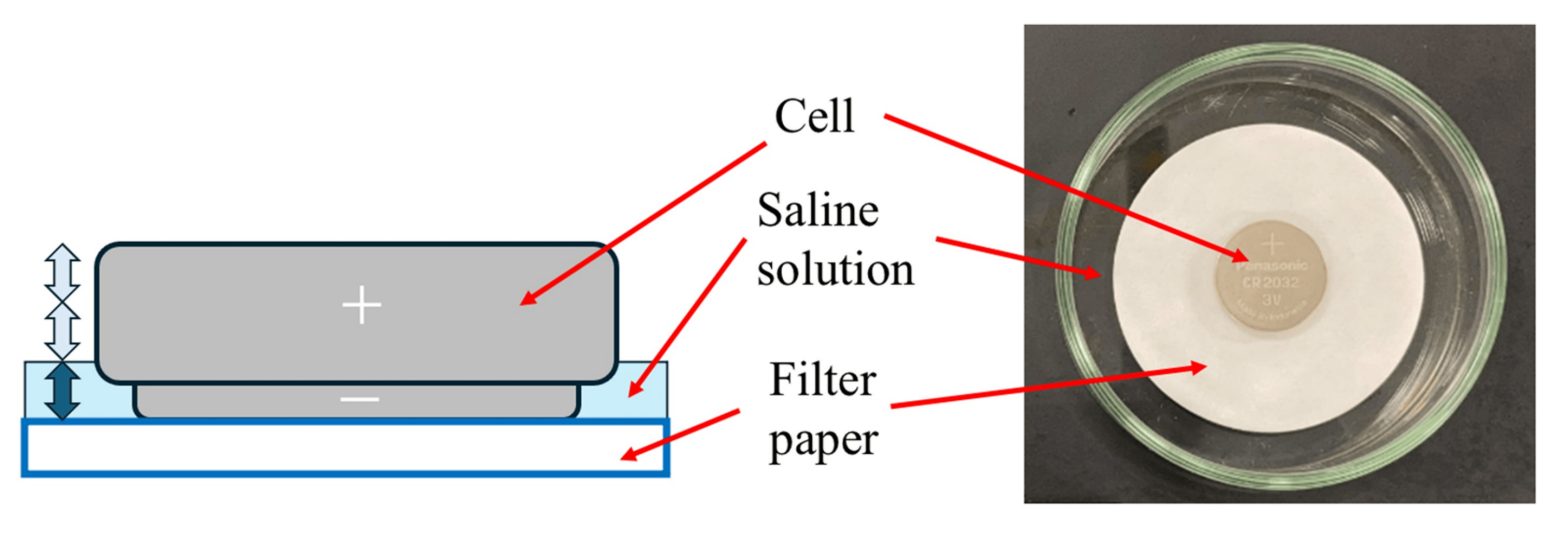
Saline soak test Schema of the saline soak test and top view at the start of the test Image Credits: Tadayoshi Takahashi

A CSLB (CR2032 model, Panasonic Energy Co. Ltd., Osaka, Japan) was placed on the filter paper with its negative side facing downward. Subsequently, 4.5 ml of saline solution was poured onto the filter paper, ensuring that the top of the positive side of the CSLB case remained dry. The Petri dish was tilted or gently moved to allow the saline to reach all parts of the dish. This volume of saline (4.5 ml) was approximately one-third of the CR2032 height (Figure 2).

A cover was then placed over the Petri dish to prevent splashing of the fluid. The CSLB was soaked in saline solution for 3 h. After 3 h, the CSLB was carefully picked up and wiped with a wet cloth, followed by a dry cloth to remove any remaining moisture on the battery surface. The CSLB was then discharged under the 15 kΩ resistor to determine its residual capacity [5]. The consumed capacity of five unused batteries was measured, and the average value was used as the initial discharge capacity. It was measured by subtracting the residual capacity of the soaked batteries from the initial discharge capacity. Three samples each of the conventional and improved batteries were tested, and their average values were subsequently compared.

In vivo experiment

All experiments were conducted in compliance with the animal experiment regulations of The Jikei University School of Medicine and in accordance with the animal experiment protocol under approval number 2019-073. The experiment was conducted using five piglets of the LWD (Landrace × Large White × Duroc) breed, each weighing approximately 14 kg. Preoperative procedures included restricting food intake from 9:00 PM the day before the experiment and limiting water intake until 2 h before the procedure. Anesthesia was induced with an intramuscular injection of medetomidine (0.06 mg/kg) and midazolam (0.3 mg/kg), followed by continuous inhalation of isoflurane for general anesthesia. The piglets were placed in a supine position with their limbs restrained, and lactated Ringer’s solution was administered intravenously through the auricular vein. Oxygen saturation and heart rate were monitored, and mechanical ventilation was performed via endotracheal intubation. A midline laparotomy and right thoracotomy were performed, followed by the dissection of the right inferior pulmonary ligament to identify the stomach and esophagus. An incision was made in the gastric body, and three unused improved CSLBs (CR2032; diameter: 20 mm, thickness: 3.2 mm, voltage: 3V, Panasonic Energy Co. Ltd., Osaka, Japan) were manually inserted and placed within the esophagus. The batteries were arranged with their positive and negative pole surfaces aligned. To prevent short-circuiting within the esophagus, a vessel loop® (Gadelius Medical K. K., Tokyo, Japan) was loosely ligated around the esophagus between the batteries. The retention times for the batteries were set at 2, 4, 6, 8, and 12 h. After each specified period, the esophagus was excised, and the piglets were euthanized with intravenous potassium chloride injection. The excised esophagus was longitudinally incised along the central axis of the battery’s positive pole surface, and the batteries were removed.

The extracted esophagus was fixed in 20% formalin solution, sectioned transversely through the center of the battery position, and histologically examined using Hematoxylin and Eosin staining. Histological tissue damage was defined as the presence of cellular necrosis, which was determined based on poor nuclear staining. The depth of tissue damage was assessed separately at the battery’s edge (the boundary between the outer case on the positive and negative poles) and the center of the negative pole surface. The depth of tissue damage was classified into the following layers: mucosal layer (from the epithelium to the submucosa), superficial muscle layer (inner circular muscle), deep muscle layer (outer longitudinal muscle), and serosa. These evaluations were conducted by a specialized pathologist. The extent of tissue damage was compared with previously reported experimental results using conventional batteries.

The discharge capacity of the batteries extracted from the piglets was measured using the same method as described in the saline soak test to assess the relationship between the in vivo discharge characteristics and simulated testing. The approximation curves were created using Excel Statistics (Microsoft Corporation, Redmond, WA, US).

## Results

Improved battery

**Figure 3:**
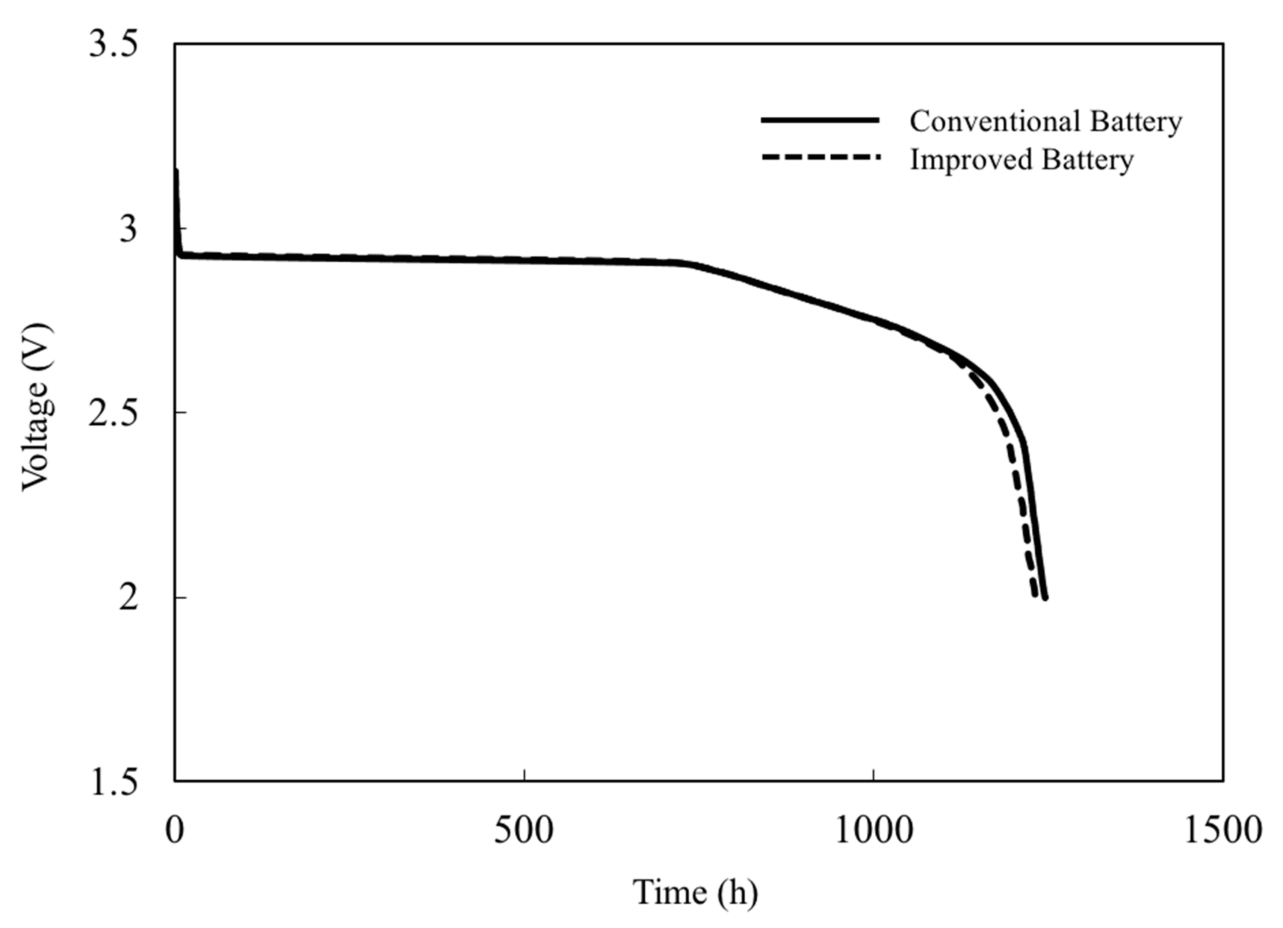
Discharge curves of conventional and improved battery Almost equivalent discharge characteristics were observed.

Visual inspection of the improved battery revealed no abnormalities in appearance, with no delamination or cracking between the layers. The improved battery exhibited discharge characteristics similar to those of conventional batteries, indicating no difference in performance (Figure 3).

In addition, concerns regarding increased contact resistance because of the titanium layer were not observed in the discharge characteristics.

Saline soak test

**Figure 4:**
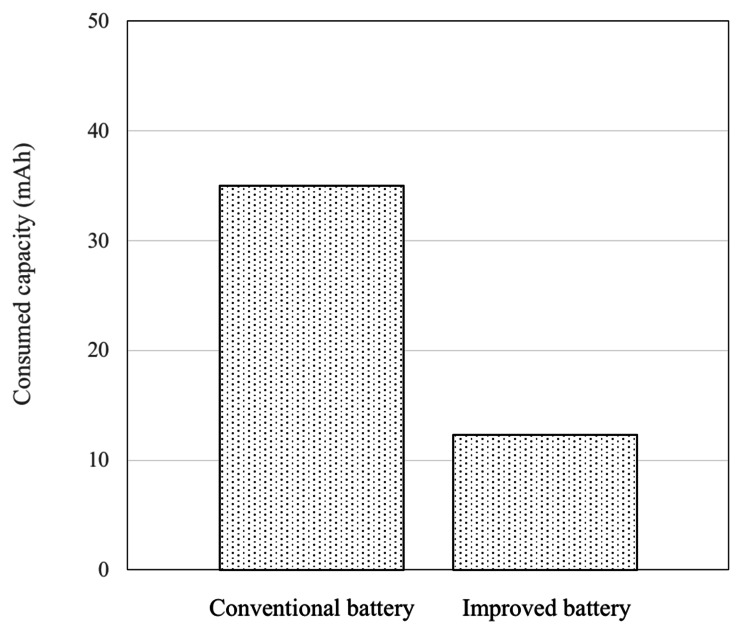
The results of the saline soak test The consumed capacity of the improved battery was almost one-third of that of the conventional battery.

In the conventional battery, the average consumed capacity after 3 h of reaction was 35 mAh. In contrast, the Ti-clad battery had an average consumed capacity of 12 mAh after 3 h. The improved battery exhibited approximately one-third of the consumed capacity compared with the conventional battery (Figure 4).

In vivo experiment

**Figure 5:**
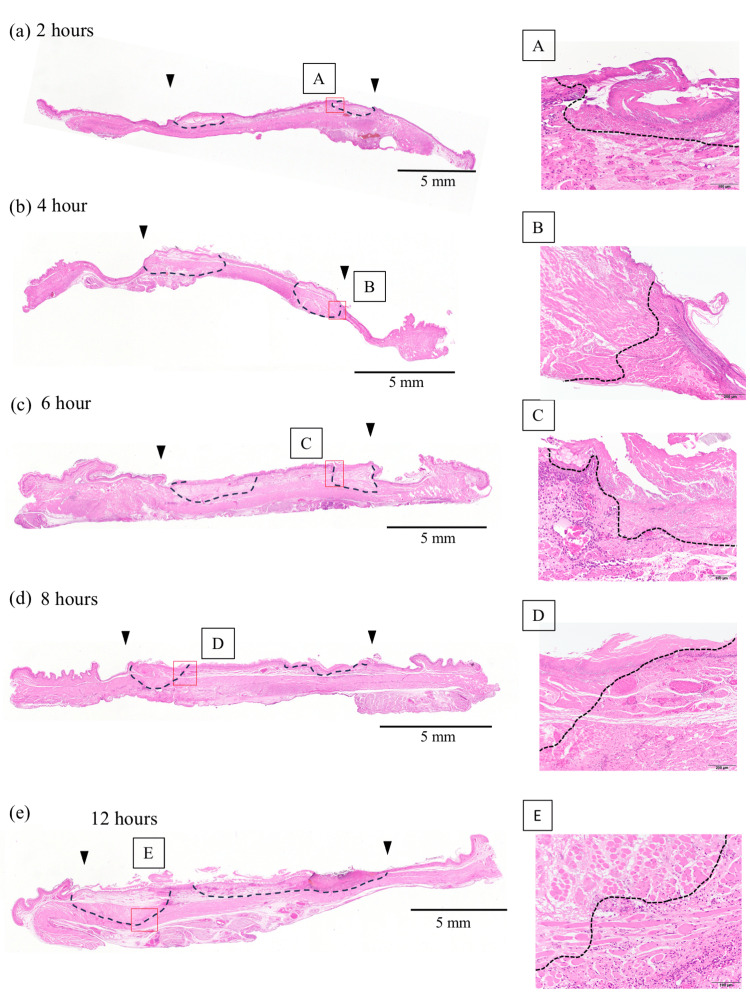
Histopathological findings (HE staining) A short-axis esophageal section is prepared at a site corresponding to the center of the battery. The boundary between the positive and negative poles is indicated by the arrowheads. The area between the black arrowheads corresponds to the negative pole. A-E are high-magnification images of the areas in the red boxes. The damage progressed both in-depth and over the entire negative pole surface. The necrotic area is indicated by a dashed line.

At the edge of the battery’s negative pole surface, tissue damage was observed in the mucosal layer after 2 h of battery placement; however, the damage did not extend to the muscle layer. Between 4 and 8 h of exposure, the tissue damage progressed, reaching into the muscle layer. After 12 h, partial full-thickness injury was observed. In contrast, at the center of the negative pole surface, no tissue damage was detected up to 8 h after battery placement. However, after 12 h, the mucosal layer exhibited damage for the first time (Figure 5). At the positive pole surface, no tissue damage was observed, consistent with findings of previous studies (Figure 5).

No unexpected bleeding, injury to other organs, hypoxia, or mortality occurred during the experiment.

**Figure 6:**
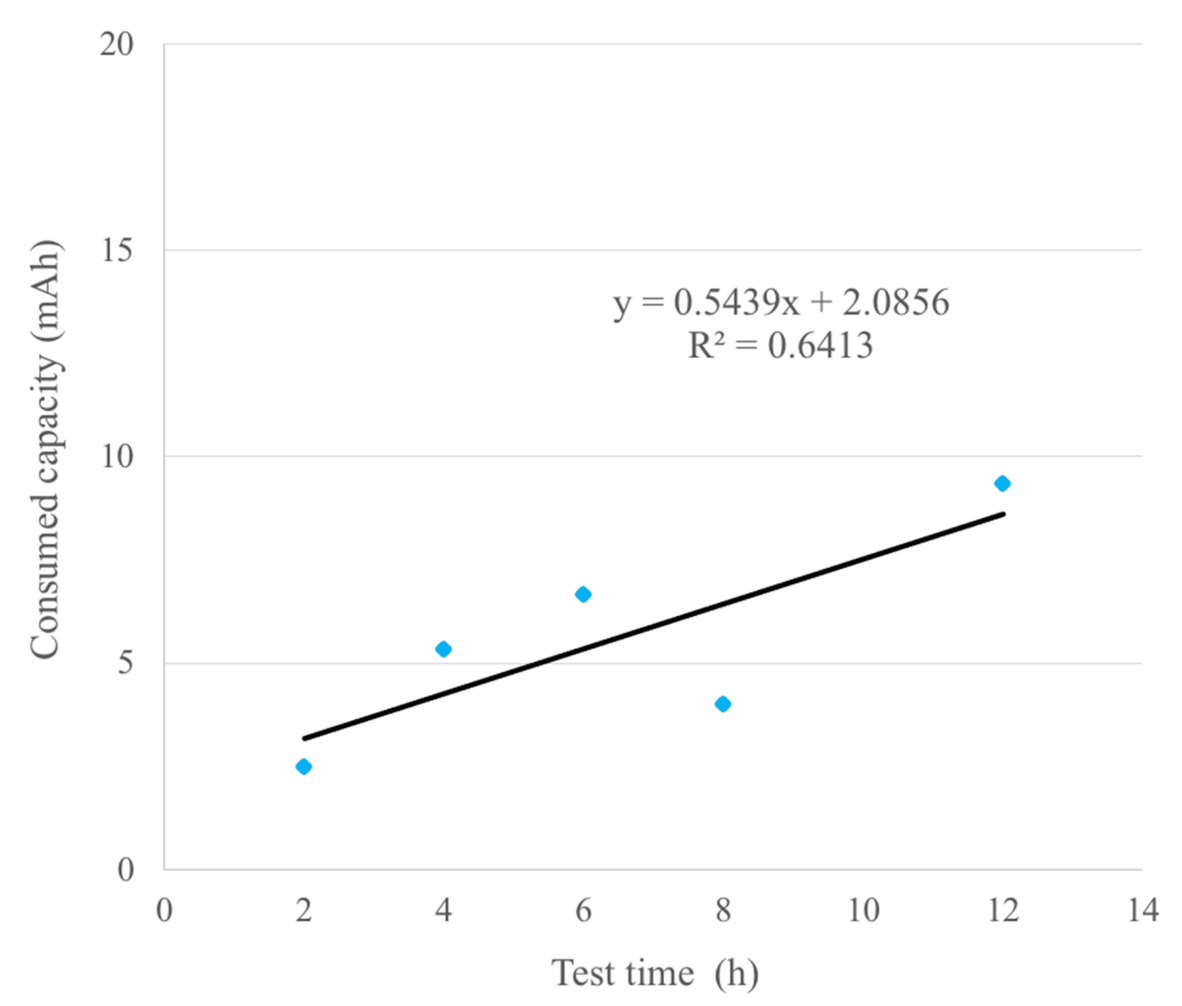
Consumed capacity of improved batteries in biological tests The average value of the consumed capacity for each test time and the approximate curve are shown.

The average consumed capacities at each experimental time point were 2.3, 5.0, 6.7, 4.0, and 9.3 mAh at 2, 4, 6, 8, and 12 h, respectively (Figure 6).

The explosive increase in consumed capacity observed in conventional batteries was not detected in the improved battery.

## Discussion

Improved battery

Conventionally, the outer cases of both the positive and negative poles of CSLBs are made of stainless steel coated with nickel plating. Upon ingestion, electrolysis of bodily fluids occurs, leading to the dissolution of nickel and stainless steel on the positive pole. Meanwhile, the negative pole undergoes water decomposition, generating alkaline ions and hydrogen gas [3]. Using titanium for the outer case of the positive pole can mitigate these reactions. However, titanium's hardness complicates bending during manufacturing, and its high cost poses additional challenges [6]. Despite this, titanium is lightweight, strong, highly corrosion-resistant, and biocompatible, making it suitable for use in artificial joints and implants. Consequently, a two-layer clad material comprising titanium and stainless steel has been proposed for the outer case of the positive pole [7]. Although this design improves the material's bendability, the titanium layer is prone to cracking, exposing the underlying stainless steel. Moreover, titanium’s natural oxide film increases contact resistance, a challenge that remains unresolved.

The advancements made in the improved battery design substantially altered the electrochemical reactions at both the positive and negative poles compared with conventional batteries. In conventional batteries, the initial dissolution process begins with the nickel-plated layer on the positive pole, followed by the dissolution of iron and other metal components from the stainless steel. The electrolysis of water on the negative pole produces hydrogen gas and alkaline hydroxide ions. In the improved battery, the reaction on the positive pole initially mirrors those of conventional batteries, with the surface nickel layer dissolving first. However, the presence of the titanium layer fundamentally alters the reaction’s dynamics; it does not dissolve and, instead, promotes oxygen gas generation through water electrolysis (Figure 1). This process is considerably slower than the dissolution of stainless steel. The reaction on the negative pole remains the same as in conventional batteries, proceeding at a very rapid rate, with the reaction at the positive pole acting as the rate-limiting step. As a result, the overall production of hydroxide ions at the negative pole is largely reduced. Furthermore, if the stainless steel were exposed at the positive pole’s edge of the outer case, conventional dissolution reactions would occur, negating the protective effects observed with the improved battery design. The insulating coating applied to the stainless steel effectively prevents this dissolution reaction, demonstrating its central role in reducing potential tissue harm.

Saline soak test

Battery ingestion is widely believed to cause tissue injury owing to electrolysis occurring between the metal outer case of the battery with the moisture from the surrounding tissues, caused by external short circuits, leading to elevated levels of alkaline hydroxide ions. Initially, we considered using meat or ham to simulate body tissues; however, due to concerns about availability and reproducibility, filter paper soaked in physiological saline solution (0.9 wt%) was chosen as the test medium. By immersing the battery in the solution and inducing an external short circuit, the discharged electric charge was measured as the battery’s discharge capacity. The amount of electric charge is proportional to the total amount of hydroxide ions generated (Figure 1). In the improved battery, the discharge capacity after 3 h was reduced to one-third of that of the conventional battery, effectively delaying tissue damage in vivo. Our previous studies established a correlation between battery discharge capacity and the depth of tissue damage [3]. This suggests that the saline soak test could serve as a potential simulation for biological responses. Notably, the improved battery exhibited a discharge capacity of 12 mAh after 3 h during the saline soak test, in contrast to an estimated capacity of 3.5 mAh calculated from the in vivo experiment approximation curve. This threefold discrepancy may stem from the continuous immersion of the battery in saline during testing, allowing the electrochemical reaction to progress without interruption, unlike in a living organism. Using the approximation curve derived from the in vivo experiments, the estimated discharge capacity at 8 h was 6.4 mAh, translating to a saline soak test capacity of 19.2 mAh. Based on these findings, future battery development should aim to maintain discharge capacities below 20 mAh in saline soak tests to mitigate the risk of fatal complications. The current saline soak test followed the IEC recommendations and produced relatively uniform results. However, the in vivo experiments exhibited variability (Figure 6). This variation is likely due to differences in esophageal diameter and moisture levels within the esophagus, which are not constant in living organisms. Consequently, although the saline soak test serves as a valuable preliminary evaluation tool, the necessity of in vivo testing remains paramount for accurately assessing actual biological responses and safety.

In vivo experiment

**Figure 7:**
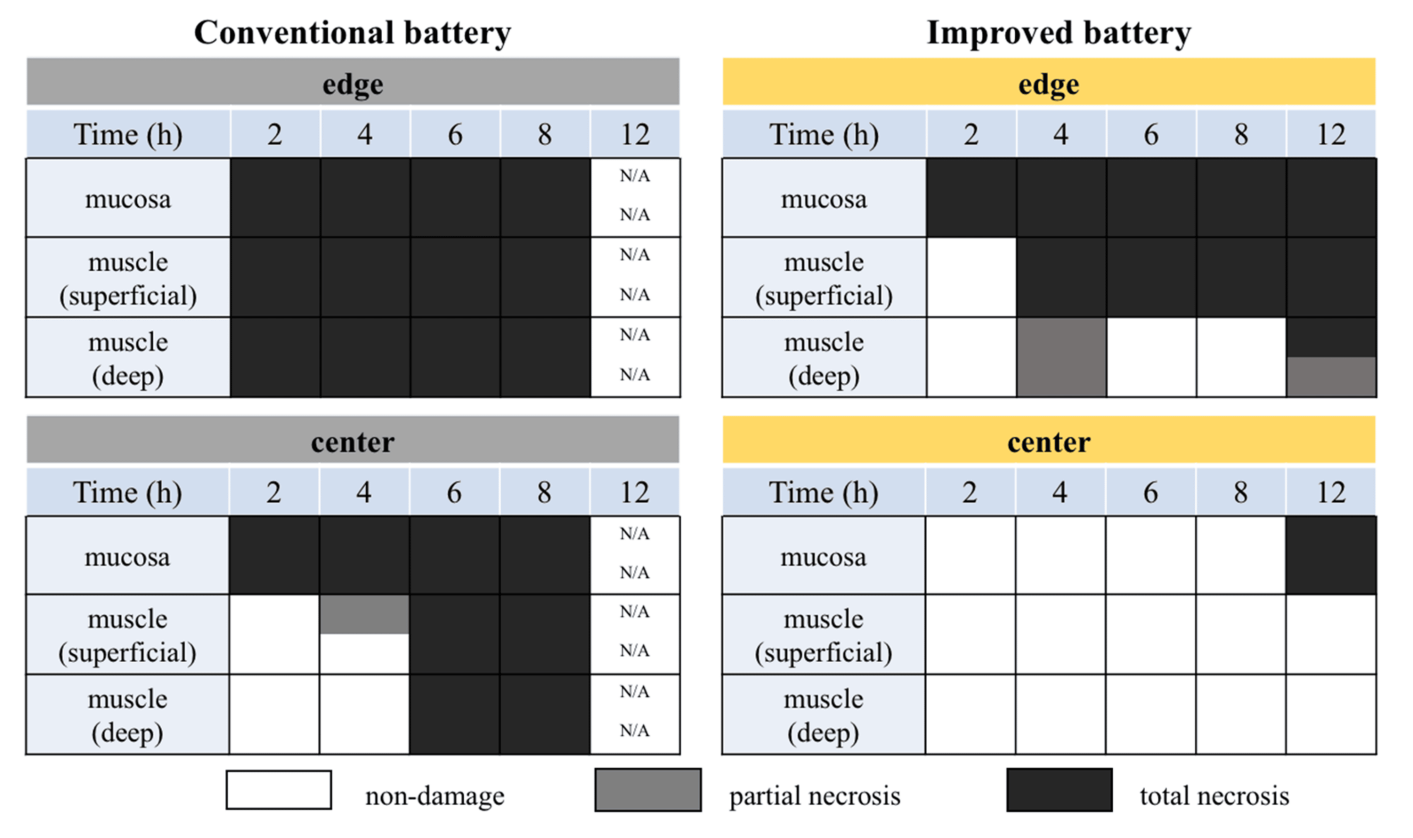
Schematic diagram of the esophageal damage over time Tissue damage progressed more slowly than in the conventional battery. In the improved battery, the full-thickness layer damage was partial even after 12 hours, indicating a response delay effect of approximately 4 to 6 times. The data for the conventional battery was taken from a previous report [3].

Compared with our previous studies on conventional batteries, tissue damage with the improved battery also originated at the edge but progressed considerably more slowly (Figure 7).

While the conventional battery caused full-thickness tissue damage within 2 h, the improved battery required 12 h with partial full-thickness damage, demonstrating a four to six-fold delay in damage progression [3]. In addition, in the conventional battery, perforation of the outer case of the positive pole was observed after 6 h, leading to rapid and severe tissue injury. In contrast, no such phenomenon was observed even after 12 h in the improved battery. This is likely due to the protective effect of the titanium layer and edge coating, which prevented the dissolution of the stainless-steel layer. Even after 12 h, the damage at the negative pole center was limited to the mucosal layer, suggesting that extensive esophageal strictures and other severe complications are unlikely. Akinkugbe reported a review of 361 pediatric cases of serious vascular complications after accidental ingestion of a button battery. They found that most of the cases with vascular complications were esophageal aortic fistulas, with a very high mortality rate of approximately 80%. In a comparison of median impaction times between survivors and non-survivors, they reported statistically significant differences at 11 h and 144 h, respectively [8]. Improvement with titanium cladding may improve the survival rate. However, some full-thickness tissue damages were observed, meaning that fatal complications, such as esophagotracheal or esophagoaortic fistulas, cannot be completely prevented. Tissue damage progresses more easily at the battery edges because the shortest distance between the positive and negative poles facilitates rapid electrochemical reactions. The improved battery incorporates a coating between the positive and negative poles, which increases this distance and likely slows the reaction rate. If this assumption holds, expanding the coating area may further delay edge damage. However, the practical application of such modifications is constrained by product design limitations.

Limitations

Individual differences in pigs, especially esophageal diameter, may affect the experimental results. To correct for individual differences, it is necessary to increase the number of samples, but this is difficult from the standpoint of animal welfare because of the large size of the animals.

The titanium cladding developed in this study requires very advanced technology. It is considered that it can be manufactured only in limited facilities.

## Conclusions

The improvements made to the battery in this study successfully extended the time required for full-thickness tissue damage at the battery edges by approximately four to six times compared with conventional batteries. This corresponds to a delay of 8-12 h, which could be critical in reducing fatal complications, particularly in countries or regions where access to timely medical care is limited. However, since the nickel layer is essential for battery function, eliminating tissue damage is not feasible.

Moreover, the cost and technical challenges associated with titanium cladding remain a substantial obstacle, highlighting the need for further innovation, including alternative approaches to battery design. Furthermore, this study demonstrated that the saline soak test can serve as a simulation for in vivo experiments, suggesting that future battery development could be accelerated by utilizing this test method.
